# Systemic inflammation and residual viraemia in HIV-positive adults on protease inhibitor monotherapy: a cross-sectional study

**DOI:** 10.1186/s12879-015-0889-9

**Published:** 2015-03-21

**Authors:** Alejandro Arenas-Pinto, Ana Milinkovic, Dimitra Peppa, Anna McKendry, Mala Maini, Richard Gilson

**Affiliations:** Centre for Sexual Health and HIV Research, Research Department of Infection and Population Health, University College London, The Mortimer Market Centre, Off Capper Street, London, WC1E 6JB UK; MRC Clinical Trials Unit at UCL, Institute of Clinical Trials & Methodology, University College London, London, UK; The Mortimer Market Centre, Central North West London NHS Foundation Trust, London, UK; Research Department of Infection and Immunity, University College London, London, UK

**Keywords:** PI monotherapy, Residual viraemia, Systemic inflammation

## Abstract

**Background:**

Increased levels of markers of systemic inflammation have been associated with serious non-AIDS events even in patients on fully suppressive antiretroviral therapy. We explored residual viremia and systemic inflammation markers in patients effectively treated with ritonavir-boosted protease inhibitor monotherapy (PImono).

**Methods:**

HIV-infected adults with persistent HIV-RNA <50 copies/ml and treated with either a) PImono or b) standard triple-drug cART were recruited for this cross-sectional, exploratory study. Plasma samples were tested for high-sensitivity CRP (hsCRP), Serum Amyloid A (SAA), soluble CD14, IL-6, IL-8 and Cytochrome C. HIV-RNA was measured by real-time PCR (detection limit of 10 copies/ml).

**Results:**

81 patients were recruited (31% on PImono). Two out of 25 (8%) and 3 of 56 (5.4%) patients from the PImono and cART groups respectively had detectable HIV-RNA. Significant correlation between SAA and hsCRP was observed (0.804). No difference between groups was found on prevalence of hsCRP >3 mg/l (21% vs 20% in the PImono and cART groups respectively; p = 0.577) or SAA >6.4 mg/l (38% vs 22% in the PImono and cART groups respectively; P = 0.172). In a univariate analysis IL6 and IL8 levels were associated with SAA >6.4 mg/l (OR = 1.74 and 1.46; 95% CI = 1.00 – 3.03 and 1.06 – 2.01; p = 0.051 and 0.02 respectively) and hsCRP >3 mg/l in (OR = 2.00 and 1.37; 95% CI = 1.09 – 3.69 and 1.02 – 1.85; p = 0.026 and 0.039 respectively).

**Conclusions:**

We found no evidence of increased levels of inflammatory biomarkers or higher prevalence of residual viraemia in patients effectively suppressed on PImono as compared with patients on standard cART.

## Background

Persistent systemic inflammation has been associated with an increased risk of serious non-AIDS defining events, such as cardiovascular disease (CVD), in HIV-positive adults despite effective treatment with antiretroviral therapy (ART) [1,2]. Standard ART regimens usually include three different drugs from at least two drug classes. However, ART simplification strategies have been proposed to reduce long-term toxicity, cost and possible pharmacokinetic interactions, particularly in an aging population that is likely to be prescribed multiple medications [3].

Protease inhibitor monotherapy (PImono) has been explored as treatment simplification strategy in a number of randomised clinical trials [4-6]. However, in clinical practice it seems like patients on PImono show a viral load rebound rate higher than what would be expected in combination ART (cART) treated patients and this has been suggested to be due to persistent residual viraemia below the detection limit of currently used assays [5]. Increased levels of systemic inflammation markers have been reported in patients with HIV low-level viraemia and have been associated with a number of serious non-AIDS defining complications. High-sensitivity C-reactive protein (hsCRP) and Interleukins 6 and 10 (IL-6, IL-10) have been reported as predictors of serious non-AIDS defining events including CVD, cancer and overall mortality [7-9]. Serum Amyloid A (SAA) is a lipoprotein secreted during the acute phase of inflammation which has been considered more sensitive than CRP in some rheumatologic conditions, although the magnitude of increase is often be greater than that of CRP [10,11]. In acute viral infections SAA levels appear to increase more than CRP [12,13].

We have performed a cross-sectional, exploratory study comparing residual viraemia and systemic inflammatory markers in patients free of major co-morbidities and effectively suppressed on either standard cART or PI monotherapy. We also aimed to explore the level of concordance between SAA and hsCRP in the study population.

## Methods

### Study population

HIV-positive adults on ART for at least 48 weeks and with documented HIV-RNA <50 copies/ml for at least 24 weeks, and no change in their ART over the same period immediately prior to enrolment were recruited for this cross-sectional study at a single site in London. At enrolment, patients were taking either a) a ritonavir-boosted protease inhibitor as a single agent (PImono) or b) cART comprising two NRTI and a third anti-retroviral agent and should have had ≥200 CD4 cell/mm^3^. Because the study aimed to investigate markers of systemic inflammation and immune activation in patients free of major co-morbidities likely to impact on these, patients were considered not eligible if they had a previous history of any autoimmune disease (e.g. systemic lupus erythematous, Addison’s disease, Graves’ disease, multiple sclerosis, myasthenia gravis, rheumatic and reactive arthritis, etc.), CVD (defined as previous myocardial infraction, stroke or coronary revascularisation), treatment for any acute opportunistic infection within three months prior to enrolment, any serious bacterial infection (e.g. pneumonia, meningitis, septicaemia, etc.) in the two months prior to enrolment, any acute infection with fever and systemic symptoms within the last 72 hours before enrolment, any vaccination in the two months prior to enrolment or if they had used systemic corticosteroids or any other immune-modulatory drug within the 12 months prior to enrolment. Patients who were hepatitis B surface antigen (HBsAg) or hepatitis C RNA positive were also excluded. Clinical and epidemiological data, including demographics, date of first HIV antibody positive result, ART history and relevant past medical history was obtained from participants medical records.

Informed consent was obtained from all study participants. The study was reviewed by the National Ethics Research Service Committee London – Queen’s Square (Ref. 11/LO/1102) and the North Central London Research Consortium R&D Office.

### Biomarkers

Plasma samples were tested for hsCRP (COBAS MIRA; Roche Diagnostics GmbH), SAA (BNII auto-analizer; Dade Behring, Marburg, Germany), sCD14, ILs1b, 6, 8, 10 and 12p70, and TNF-α (BD Biosciences CBA, San Jose, CA, USA) and Cytochrome C (R&D Quantikine, MN, USA). HIV-RNA was measured by real-time PCR with a detection limit of 10 copies/ml (in-house developed assay by UCLH virology department; CPA accredited). Samples were stored at −80°C and tested at the end of the study period in a single batch.

### Statistical analysis

Continuous data were summarised and compared using the Mann-Witney test or grouped into categories where Fischer’s exact test was used. Significance level set as p < 0.05 in all analyses. Clinically significant elevation of hsCRP and SAA were defined as >3 and 6.4 mg/l respectively. Simple linear regression was used to explore correlation between hsCRP and SAA levels. Associations with elevated hsCRP and SAA were explored using logistic regression.

## Results

A total of 81 treated HIV-infected patients were recruited of whom, 25 (31%) were being treated with PImono. Most of patients on cART were receiving abacavir-containing (96%) and PI-based (57%) combinations. Patients on PImono were mainly receiving DRV/RTV (80%) (Table 1). Median age was 46.2 years (Inter quartile range (IQR) 42.2 – 52.7) and there was no difference between study groups on age distribution. There were more female patients in the cART group (26.8%) compared to the PImono group (4%; p = 0.017). Patients on cART were known with HIV infection for slightly longer (median 11.8; IQR = 8.2-16.7 years) than patients on PImono (median 9.6; IQR = 7.3- 14.3 years) (p = 0.091). Median CD4 count was similar between study groups (550 cell/ml; IQR = 460-740 and 615 cell/ml; IQR = 470-770 for the PImono and the cART groups respectively; p = 0.210). All study participants had HIV-RNA <50 copies/ml at entry. Two out of 25 (8%) and 3 of 56 (5.4%) patients from the PImono and cART groups respectively had residual viraemia (i.e. HIV-RNA 10–50 copies/ml) (p = 0.648). Two of the patients with detectable viraemia were being treated with ritonavir-boosted darunavir monotherapy whereas the other three were on abacavir, lamivudine and either efavirenz or nevirapine.

**Table 1:** **Patients characteristics**

	**PI Monotherapy (N = 25)**		**cART (N = 56)**		**P** ^**1**^
	**N**	**%**	**N**	**%**	
Female sex	1	4.0	15	26.8	0.017
Detectable HIV-RNA^2^	2	8.0	3	5.4	0.648
hsCRP >3 mg/l	5	20.8	11	20.0	0.577
SAA >6.4 mg/l	9	37.5	12	21.82	0.172
IL6 > 3 pg/ml	1	4.8	1	1.9	0.490
PI exposure	25	100.0	32	57.0	
ATV/RTV	3	12.0	16	28.6	
DRV/RTV	20	80.0	9	16.1	
LPV/RTV	2	8.0	4	7.1	
Other PI	0	0.0	3	5.4	
NNRTI exposure			23	41.1	
EFV			11	19.6	
NVP			12	21.4	
NRTI exposure			56	100.0	
ABC			54	96.4	
TDF			5	8.9	

^1^Fisher’s exact test.

^2^higher than 10 copies/ml.

There was no difference in the distribution of hsCRP and SAA levels between the study groups (Table 2). Overall, 20% of participants had hsCRP >3 mg/l, but no difference between groups was found (21% vs 20% in the PImono and cART groups respectively; p = 0.577). Similarly, there was no difference between groups in the proportion of participants with SAA >6.4 mg/l (upper limit of normal range) (38% vs 22% in the PImono and cART groups respectively; p = 0.172). We found no difference on the proportion of patients with elevated hsCRP or SAA between patients on PImono or NNRTI-based cART (p = 0.702 and 0.096 respectively). Other markers of inflammation and mitochondrial function did not differ between groups as shown in Table 2.

**Table 2:** **Systemic inflammation and mitochondrial dysfunction markers**

	**PI Monotherapy (N = 25)**		**cART (N = 56)**		P*	**Overall (N = 81)**	
	**Median**	**IQR**	**Median**	**IQR**		**Median**	**IQR**
hsCRP (mg/l)	0.85	0.54 - 2.135	0.96	0.56 - 2.44	0.798	0.93	0.56 - 2.41
SAA (mg/l)	1.8	1.35 - 9.3	3.5	1.8 - 3.5	0.462	3.1	1.6 - 6.8
IL-6 pg/ml	1.51	1.28 - 2.95	1.41	1.21 - 1.86	0.675	1.45	1.24 - 1.87
IL-8 pg/ml	2.39	1.84 - 3.9	2.56	1.94 - 3.34	0.792	2.54	1.92 - 3.48
Cytochrome C (ng/dl)	3.444	2.596 - 4.449	3.349	2.11 - 4.593	0.575	3.444	2.219 - 4.59
sCD14 (ng/dl)	1.744	1.575 - 2.071	1.758	1.5 - 2.289	0.783	1.744	1.511 - 2.147

*Mann–Whitney test.

A significant correlation between SAA and hsCRP was observed (0.804) with a regression coefficient of 3.507 (95% CI 2.92-4.20; p < 0.001) suggesting an expected increment of 3 mg/l in SAA for each unit increment in hsCRP. A total of 14 participants had hsCRP >3 mg/l and SAA >6.4 mg/l, whereas seven of the 21 patients with SAA >6.4 mg/l (33%) had hsCRP <3 mg/l. In a logistic regression analysis IL6 and IL8 levels were associated with SAA >6.4 mg/l (OR = 1.74 and 1.46; 95% CI 1.00-3.03 and 1.06-2.01; p = 0.051 and 0.02 respectively) whereas younger age and female gender showed an inverse association with SAA >6.4 mg/l (OR = 0.94 and 0.37; 95% CI 0.88-1.00 and 0.12-1.16 respectively). In a multivariate model only age remained independently associated with SAA >6.4 mg/l (OR = 0.92; 95% CI 0.86-0.99; p = 0.038). IL6 and IL8 levels were also associated with hsCRP >3 mg/l in univariate analysis (OR = 2.00 and 1.37; 95% CI 1.09-3.69 and 1.02-1.85; p = 0.026 and 0.039 respectively).

## Discussion

We did not find any evidence of increased levels of inflammatory biomarkers or higher prevalence of residual viraemia in patients with HIV viral loads <50 copies/ml while treated with PImono as compared with patients on cART which is consistent with previous studies where PI monotherapy has been compared to PI-based cART [14,15]. However, Torres et al. have reported higher monocyte activation, inflammation and residual viraemia in patients on PI monotherapy compared to PI-based cART [16]. In our study the majority of subjects showed low plasma levels of hsCRP and SAA as expected in well suppressed patients with no major co-morbidities. However, the levels of CRP and IL6 reported in previous studies with less stringent exclusion criteria were much higher and the proportion of participants with known co-morbidities such as hepatitis C was about 20% [15,16]. Increased monocyte activation, systemic inflammation and immune activation markers have all been associated with a number of inflammatory conditions, including HCV infection [17], and some of these could have been contributed to partially explain controversial results.

Residual viraemia has been observed more frequently in patients on PI-based compared to NNRTI-based cART [18]. However, in our study 40% of the control group were on NNRT-base cART and we still found no difference in residual viraemia between groups. In randomised trials PI monotherapy has been associated with a higher viral load rebound rate compared to cART but ours and previous results appear to suggest that persistent residual viraemia may not be the mechanism for viral load rebounds in patients treated with PI as single agent [4-6].

Because the detection limit of the PCR assay we used, we cannot exclude the possibility of differences in very low level viraemia (i.e. <10 copies/ml) between groups, but if there was any, that does not appeared to have any impact on systemic inflammation. However, we found that 26% of the study population had SAA levels >6.4 mg/l, which are considered elevated, and have been associated with active inflammatory conditions [19]. Furthermore, clinically significant elevations in SAA were associated with IL-6 and IL-8 levels and these have been previously reported to be associated with serious non-AIDS events [20]. Further investigation of SAA as a marker of systemic inflammation in HIV-infected populations may be of interest.

Mitochondrial dysfunction may lead to systemic inflammation and has been reported as an important mechanism in acute response to trauma [21]. NRTIs can induce mitochondrial dysfunction and this could be a mechanism to trigger an inflammatory response locally or systemically. Cytochrome-C (Cyt-C) is a marker of mitochondrial function and integrity and has been associated with inflammation and apoptosis [22] but, in our study, plasma levels of Cyt-C were not different between groups. Most of participants in the cART group (96%) were taking abacavir-based ART which is less likely to cause mitochondrial impairment than other NRTI although it has been associated with an excess risk for cardiovascular events [23,24].

Our study has limitations related to the study design and the small size of the study population. In addition, the study population included a selected group of patients and treatment allocation was not randomised. However, we believe ours is the first study looking at the question of systemic inflammation and low-level viraemia in patients on PI monotherapy compared to PI and NNRTI-based cART in a population free of serious co-morbidities, meaning that the effect of any concomitant morbidity on systemic inflammation can be ruled out.

## Conclusions

Our data add further evidence suggesting that patients with persistent HIV-RNA <50 copies/ml while on PI monotherapy are not more likely to have increased levels of systemic inflammation markers circulating in plasma or residual viraemia than patients on standard cART.

## Abbreviations

ART/cART: Anti-retroviral therapy/Combination Anti-retroviral therapy
CRP/hsCRP: C-Reactive Protein/High-sensitivity C-Reactive Protein
CVD: Cardiovascular disease
Cyt-C: Cytochrome-C
IL: Interleukin
NRTI/NNRTI: Nucleoside reverse transcriptase inhibitor/Non-nucleoside reverse transcriptase inhibitor
PI/PImono: Protease Inhibitor/Protease Inhibitor monotherapy
SSA: Serum Amyloid A
